# (2,3-Di-2-pyridyl­pyrazine-κ^2^ *N* ^2^,*N* ^3^)diiodidopalladium(II)

**DOI:** 10.1107/S1600536811044023

**Published:** 2011-10-29

**Authors:** Kwang Ha

**Affiliations:** a School of Applied Chemical Engineering, The Research Institute of Catalysis, Chonnam National University, Gwangju 500-757, Republic of Korea

## Abstract

The Pd^II^ ion in the title complex, [PdI_2_(C_14_H_10_N_4_)], is four-coordinated in a slightly distorted square-planar environment by the two pyridine N atoms of the chelating 2,3-di-2-pyridyl­pyrazine (dpp) ligand and two iodide anions. In the crystal, the pyridine rings are considerably inclined to the least-squares plane of the PdI_2_N_2_ unit [maximum deviation = 0.090 (2) Å], making dihedral angles of 65.0 (2) and 66.6 (2)°. The pyrazine ring is perpendicular to the unit plane, with a dihedral angle of 89.9 (2)°. The complex mol­ecules are connected by C—H⋯I hydrogen bonds, forming a helical chain along the *b* axis.

## Related literature

For the crystal structure of the yellow form of [PtBr_2_(dpp)] which is isotypic to the title complex, see: Ha (2011 ▶).
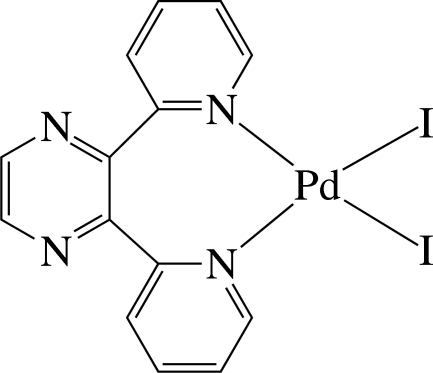


## Experimental

### 

#### Crystal data

[PdI_2_(C_14_H_10_N_4_)]
*M* *_r_* = 594.46
Monoclinic, 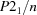

*a* = 8.7936 (12) Å
*b* = 15.528 (2) Å
*c* = 12.3351 (17) Å
β = 102.118 (3)°
*V* = 1646.8 (4) Å^3^
*Z* = 4
Mo *K*α radiation
μ = 4.87 mm^−1^
*T* = 200 K
0.42 × 0.31 × 0.29 mm

#### Data collection

Bruker SMART 1000 CCD diffractometer
Absorption correction: multi-scan (*SADABS*; Bruker, 2000 ▶) *T*_min_ = 0.183, *T*_max_ = 0.243
10659 measured reflections
3513 independent reflections
2968 reflections with *I* > 2σ(*I*)
*R*_int_ = 0.022

#### Refinement

*R*[*F* ^2^ > 2σ(*F* ^2^)] = 0.035
*wR*(*F* ^2^) = 0.103
*S* = 1.05
3513 reflections
190 parameters
H-atom parameters constrained
Δρ_max_ = 1.28 e Å^−3^
Δρ_min_ = −1.42 e Å^−3^

### 

Data collection: *SMART* (Bruker, 2000 ▶); cell refinement: *SAINT* (Bruker, 2000 ▶); data reduction: *SAINT*; program(s) used to solve structure: *SHELXS97* (Sheldrick, 2008 ▶); program(s) used to refine structure: *SHELXL97* (Sheldrick, 2008 ▶); molecular graphics: *ORTEP-3* (Farrugia, 1997 ▶) and *PLATON* (Spek, 2009 ▶); software used to prepare material for publication: *SHELXL97*.

## Supplementary Material

Crystal structure: contains datablock(s) global, I. DOI: 10.1107/S1600536811044023/is2795sup1.cif

Structure factors: contains datablock(s) I. DOI: 10.1107/S1600536811044023/is2795Isup2.hkl

Additional supplementary materials:  crystallographic information; 3D view; checkCIF report

## Figures and Tables

**Table 1:** Selected bond lengths (Å)

Pd1—N3	2.050 (5)
Pd1—N4	2.056 (5)
Pd1—I1	2.5761 (7)
Pd1—I2	2.5898 (6)

**Table 2:** Hydrogen-bond geometry (Å, °)

*D*—H⋯*A*	*D*—H	H⋯*A*	*D*⋯*A*	*D*—H⋯*A*
C11—H11⋯I1^i^	0.95	2.99	3.776 (7)	141

Symmetry code: (i) 
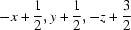
.

**Fig. 1.**
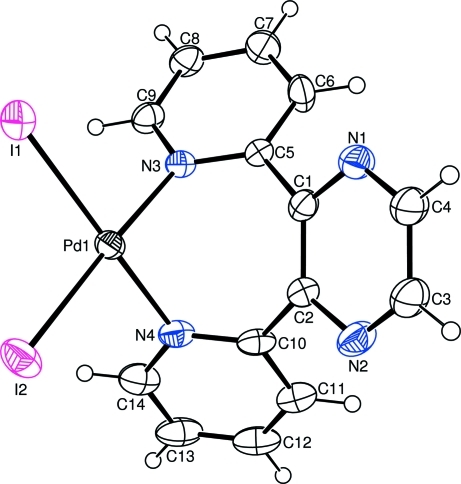
The structure of the title complex, with displacement ellipsoids drawn at the 40% probability level; H atoms are shown as small circles of arbitrary radius.

**Fig. 2.**
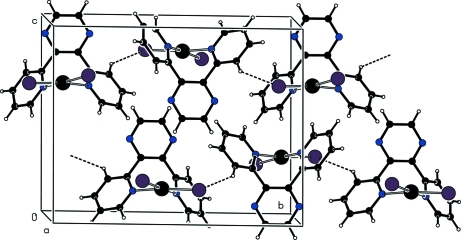
View of the hydrogen-bond interactions of the title complex. Hydrogen-bonds are drawn with dashed lines.

## supplementary crystallographic information

### Comment

The title complex, [PdI_2_(dpp)] (dpp is 2,3-di-2-pyridylpyrazine, C_14_H_10_N_4_), is isomorphous with the yellow form of [PtBr_2_(dpp)] (Ha, 2011). The Pd^II^ ion is four-coordinated in a slightly distorted square-planar environment by the two pyridyl N atoms of the chelating dpp ligand and two iodide anions (Fig. 1). The contributions to the distortion are the N3—Pd1—N4 chelate angle of 87.17 (19)° and I—I repelling, and therefore the *trans* axes are slightly bent [<I1—Pd1—N4 = 173.87 (13)° and <I2—Pd1—N3 = 176.70 (13)°]. The Pd—N and Pd—I bond lengths are nearly equivalent, respectively (Table 1). In the crystal, the two pyridyl rings are considerably inclined to the least-squares plane of the PdI_2_N_2_ unit [maximum deviation = 0.090 (2) Å] with dihedral angles of 65.0 (2) and 66.6 (2)°, respectively. The nearly planar pyrazine ring [maximum deviation = 0.014 (4) Å] is perpendicular to the unit plane with a dihedral angle of 89.9 (2)°. The dihedral angle between the two pyridyl rings is 78.6 (2)°. The complexes are connected by C—H···I hydrogen bonds, forming a helical chain running along the *b* axis (Fig. 2 and Table 2), and stacked in columns along the *a* axis. When viewed down the *b* axis, the successive complexes stack in the opposite direction. In the columns, numerous inter- and intramolecular π–π interactions between the six-membered rings are present, the shortest ring centroid-centroid distance being 3.969 (4) Å.

### Experimental

To a solution of Na_2_PdCl_4_ (0.1464 g, 0.498 mmol) and KI (0.7790 g, 4.693 mmol) in MeOH (30 ml) was added 2,3-di-2-pyridylpyrazine (0.1183 g, 0.505 mmol) and stirred for 3 h at room temperature. The formed precipitate was separated by filtration, washed with MeOH and acetone, and dried at 50 °C, to give a redbrown powder (0.2537 g). Crystals suitable for X-ray analysis were obtained by slow evaporation from a CH_3_CN solution.

### Refinement

H atoms were positioned geometrically and allowed to ride on their respective parent atoms [C—H = 0.95 Å and *U*_iso_(H) = 1.2*U*_eq_(C)]. The highest peak (1.27 e Å^-3^) and the deepest hole (-1.41 e Å^-3^) in the difference Fourier map are located 0.72 Å and 0.59 Å from the atoms I2 and I1, respectively.

### Crystal data

**Table:**

[PdI_2_(C_14_H_10_N_4_)]	*F*(000) = 1096
*M**_r_* = 594.46	*D*_x_ = 2.398 Mg m^−^^3^
Monoclinic, *P*2_1_/*n*	Mo *K*α radiation, λ = 0.71073 Å
Hall symbol: -P 2yn	Cell parameters from 7148 reflections
*a* = 8.7936 (12) Å	θ = 2.4–27.0°
*b* = 15.528 (2) Å	µ = 4.87 mm^−^^1^
*c* = 12.3351 (17) Å	*T* = 200 K
β = 102.118 (3)°	Block, red-brown
*V* = 1646.8 (4) Å^3^	0.42 × 0.31 × 0.29 mm
*Z* = 4	

### Data collection

**Table:**

Bruker SMART 1000 CCD diffractometer	3513 independent reflections
Radiation source: fine-focus sealed tube	2968 reflections with *I* > 2σ(*I*)
graphite	*R*_int_ = 0.022
φ and ω scans	θ_max_ = 27.0°, θ_min_ = 2.1°
Absorption correction: multi-scan (*SADABS*; Bruker, 2000)	*h* = −11→10
*T*_min_ = 0.183, *T*_max_ = 0.243	*k* = −19→19
10659 measured reflections	*l* = −11→15

### Refinement

**Table:**

Refinement on *F*^2^	Primary atom site location: structure-invariant direct methods
Least-squares matrix: full	Secondary atom site location: difference Fourier map
*R*[*F*^2^ > 2σ(*F*^2^)] = 0.035	Hydrogen site location: inferred from neighbouring sites
*wR*(*F*^2^) = 0.103	H-atom parameters constrained
*S* = 1.05	*w* = 1/[σ^2^(*F*_o_^2^) + (0.0477*P*)^2^ + 7.4036*P*] where *P* = (*F*_o_^2^ + 2*F*_c_^2^)/3
3513 reflections	(Δ/σ)_max_ = 0.001
190 parameters	Δρ_max_ = 1.28 e Å^−^^3^
0 restraints	Δρ_min_ = −1.42 e Å^−^^3^

### Special details

**Table:**

Geometry. All e.s.d.'s (except the e.s.d. in the dihedral angle between two l.s. planes) are estimated using the full covariance matrix. The cell e.s.d.'s are taken into account individually in the estimation of e.s.d.'s in distances, angles and torsion angles; correlations between e.s.d.'s in cell parameters are only used when they are defined by crystal symmetry. An approximate (isotropic) treatment of cell e.s.d.'s is used for estimating e.s.d.'s involving l.s. planes.
Refinement. Refinement of *F*^2^ against ALL reflections. The weighted *R*-factor *wR* and goodness of fit *S* are based on *F*^2^, conventional *R*-factors *R* are based on *F*, with *F* set to zero for negative *F*^2^. The threshold expression of *F*^2^ > σ(*F*^2^) is used only for calculating *R*-factors(gt) *etc*. and is not relevant to the choice of reflections for refinement. *R*-factors based on *F*^2^ are statistically about twice as large as those based on *F*, and *R*- factors based on ALL data will be even larger.

### Fractional atomic coordinates and isotropic or equivalent isotropic displacement parameters (Å^2^)

**Table:**

	*x*	*y*	*z*	*U*_iso_*/*U*_eq_	
Pd1	0.46673 (5)	0.06358 (3)	0.67952 (3)	0.03147 (13)	
I1	0.61860 (5)	−0.07875 (3)	0.68473 (4)	0.04827 (15)	
I2	0.71972 (6)	0.15314 (3)	0.73389 (4)	0.05207 (16)	
N1	0.1852 (6)	0.0054 (3)	0.9114 (4)	0.0419 (12)	
N2	0.2351 (7)	0.1813 (4)	0.9159 (5)	0.0473 (13)	
N3	0.2628 (5)	−0.0048 (3)	0.6446 (4)	0.0314 (10)	
N4	0.3330 (6)	0.1733 (3)	0.6587 (4)	0.0375 (11)	
C1	0.2007 (6)	0.0473 (4)	0.8187 (5)	0.0332 (12)	
C2	0.2265 (7)	0.1360 (4)	0.8211 (5)	0.0347 (12)	
C3	0.2229 (9)	0.1385 (5)	1.0064 (6)	0.0516 (17)	
H3	0.2304	0.1690	1.0740	0.062*	
C4	0.1993 (9)	0.0498 (4)	1.0047 (6)	0.0495 (16)	
H4	0.1932	0.0207	1.0714	0.059*	
C5	0.1683 (6)	−0.0075 (3)	0.7174 (5)	0.0309 (11)	
C6	0.0425 (7)	−0.0623 (4)	0.7030 (6)	0.0430 (15)	
H6	−0.0216	−0.0642	0.7561	0.052*	
C7	0.0103 (7)	−0.1154 (4)	0.6092 (6)	0.0485 (16)	
H7	−0.0753	−0.1541	0.5981	0.058*	
C8	0.1045 (7)	−0.1106 (4)	0.5334 (5)	0.0418 (14)	
H8	0.0844	−0.1452	0.4684	0.050*	
C9	0.2272 (7)	−0.0552 (4)	0.5534 (5)	0.0385 (13)	
H9	0.2913	−0.0517	0.5004	0.046*	
C10	0.2329 (7)	0.1913 (4)	0.7239 (5)	0.0374 (13)	
C11	0.1398 (8)	0.2639 (4)	0.7055 (6)	0.0470 (16)	
H11	0.0711	0.2768	0.7533	0.056*	
C12	0.1467 (10)	0.3171 (4)	0.6187 (6)	0.0558 (19)	
H12	0.0820	0.3665	0.6050	0.067*	
C13	0.2478 (10)	0.2984 (4)	0.5515 (6)	0.0554 (19)	
H13	0.2543	0.3348	0.4907	0.067*	
C14	0.3406 (9)	0.2259 (4)	0.5733 (5)	0.0497 (16)	
H14	0.4113	0.2130	0.5270	0.060*	

### Atomic displacement parameters (Å^2^)

**Table:**

	*U*^11^	*U*^22^	*U*^33^	*U*^12^	*U*^13^	*U*^23^
Pd1	0.0364 (2)	0.0300 (2)	0.0295 (2)	−0.00616 (17)	0.01056 (17)	−0.00251 (16)
I1	0.0419 (3)	0.0503 (3)	0.0548 (3)	0.00162 (18)	0.01512 (19)	0.00031 (19)
I2	0.0562 (3)	0.0592 (3)	0.0431 (3)	−0.0268 (2)	0.0154 (2)	−0.0105 (2)
N1	0.053 (3)	0.037 (3)	0.036 (3)	−0.003 (2)	0.013 (2)	−0.002 (2)
N2	0.062 (4)	0.039 (3)	0.041 (3)	0.002 (3)	0.012 (3)	−0.011 (2)
N3	0.034 (2)	0.026 (2)	0.034 (2)	−0.0009 (18)	0.0070 (19)	0.0001 (19)
N4	0.054 (3)	0.027 (2)	0.031 (2)	−0.004 (2)	0.007 (2)	−0.0016 (19)
C1	0.032 (3)	0.034 (3)	0.034 (3)	0.001 (2)	0.007 (2)	−0.006 (2)
C2	0.038 (3)	0.030 (3)	0.037 (3)	−0.001 (2)	0.010 (2)	−0.004 (2)
C3	0.072 (5)	0.048 (4)	0.038 (4)	−0.002 (3)	0.018 (3)	−0.009 (3)
C4	0.068 (5)	0.044 (4)	0.040 (4)	−0.001 (3)	0.019 (3)	−0.004 (3)
C5	0.034 (3)	0.026 (3)	0.034 (3)	0.001 (2)	0.010 (2)	−0.003 (2)
C6	0.034 (3)	0.055 (4)	0.044 (4)	−0.009 (3)	0.017 (3)	−0.009 (3)
C7	0.037 (3)	0.046 (4)	0.062 (4)	−0.012 (3)	0.008 (3)	−0.014 (3)
C8	0.047 (4)	0.038 (3)	0.039 (3)	−0.005 (3)	0.004 (3)	−0.014 (3)
C9	0.048 (3)	0.037 (3)	0.031 (3)	0.000 (3)	0.008 (2)	−0.007 (2)
C10	0.050 (3)	0.026 (3)	0.033 (3)	−0.004 (2)	−0.001 (3)	−0.003 (2)
C11	0.053 (4)	0.033 (3)	0.052 (4)	0.003 (3)	0.003 (3)	−0.004 (3)
C12	0.069 (5)	0.029 (3)	0.060 (4)	0.000 (3)	−0.008 (4)	0.004 (3)
C13	0.092 (6)	0.032 (3)	0.036 (3)	−0.007 (3)	0.001 (4)	0.004 (3)
C14	0.071 (5)	0.041 (3)	0.036 (3)	−0.011 (3)	0.008 (3)	−0.001 (3)

### Geometric parameters (Å, °)

**Table:**

Pd1—N3	2.050 (5)	C4—H4	0.9500
Pd1—N4	2.056 (5)	C5—C6	1.378 (8)
Pd1—I1	2.5761 (7)	C6—C7	1.400 (9)
Pd1—I2	2.5898 (6)	C6—H6	0.9500
N1—C4	1.326 (8)	C7—C8	1.375 (10)
N1—C1	1.347 (8)	C7—H7	0.9500
N2—C3	1.321 (9)	C8—C9	1.362 (9)
N2—C2	1.353 (8)	C8—H8	0.9500
N3—C5	1.346 (7)	C9—H9	0.9500
N3—C9	1.352 (7)	C10—C11	1.384 (9)
N4—C10	1.340 (8)	C11—C12	1.363 (10)
N4—C14	1.346 (8)	C11—H11	0.9500
C1—C2	1.395 (8)	C12—C13	1.367 (12)
C1—C5	1.489 (7)	C12—H12	0.9500
C2—C10	1.486 (8)	C13—C14	1.383 (11)
C3—C4	1.393 (10)	C13—H13	0.9500
C3—H3	0.9500	C14—H14	0.9500
			
N3—Pd1—N4	87.17 (19)	C6—C5—C1	117.9 (5)
N3—Pd1—I1	89.26 (13)	C5—C6—C7	119.2 (6)
N4—Pd1—I1	173.87 (13)	C5—C6—H6	120.4
N3—Pd1—I2	176.70 (13)	C7—C6—H6	120.4
N4—Pd1—I2	91.43 (14)	C8—C7—C6	118.9 (6)
I1—Pd1—I2	92.38 (2)	C8—C7—H7	120.5
C4—N1—C1	118.5 (5)	C6—C7—H7	120.5
C3—N2—C2	118.0 (6)	C9—C8—C7	118.6 (6)
C5—N3—C9	118.0 (5)	C9—C8—H8	120.7
C5—N3—Pd1	120.7 (4)	C7—C8—H8	120.7
C9—N3—Pd1	120.9 (4)	N3—C9—C8	123.6 (6)
C10—N4—C14	119.3 (6)	N3—C9—H9	118.2
C10—N4—Pd1	121.9 (4)	C8—C9—H9	118.2
C14—N4—Pd1	118.7 (5)	N4—C10—C11	120.8 (6)
N1—C1—C2	120.3 (5)	N4—C10—C2	120.3 (5)
N1—C1—C5	113.6 (5)	C11—C10—C2	118.9 (6)
C2—C1—C5	125.7 (5)	C12—C11—C10	120.0 (7)
N2—C2—C1	120.7 (6)	C12—C11—H11	120.0
N2—C2—C10	113.1 (5)	C10—C11—H11	120.0
C1—C2—C10	126.0 (5)	C11—C12—C13	119.3 (7)
N2—C3—C4	121.5 (6)	C11—C12—H12	120.4
N2—C3—H3	119.2	C13—C12—H12	120.4
C4—C3—H3	119.2	C12—C13—C14	119.2 (7)
N1—C4—C3	120.9 (6)	C12—C13—H13	120.4
N1—C4—H4	119.5	C14—C13—H13	120.4
C3—C4—H4	119.5	N4—C14—C13	121.4 (7)
N3—C5—C6	121.6 (5)	N4—C14—H14	119.3
N3—C5—C1	120.4 (5)	C13—C14—H14	119.3
			
N4—Pd1—N3—C5	−70.8 (4)	N1—C1—C5—C6	42.3 (7)
I1—Pd1—N3—C5	114.2 (4)	C2—C1—C5—C6	−130.1 (7)
N4—Pd1—N3—C9	116.0 (4)	N3—C5—C6—C7	−1.3 (10)
I1—Pd1—N3—C9	−59.1 (4)	C1—C5—C6—C7	−179.2 (6)
N3—Pd1—N4—C10	62.2 (5)	C5—C6—C7—C8	−0.6 (11)
I2—Pd1—N4—C10	−114.8 (4)	C6—C7—C8—C9	1.0 (10)
N3—Pd1—N4—C14	−114.1 (5)	C5—N3—C9—C8	−2.4 (9)
I2—Pd1—N4—C14	68.9 (5)	Pd1—N3—C9—C8	171.0 (5)
C4—N1—C1—C2	−1.8 (9)	C7—C8—C9—N3	0.6 (10)
C4—N1—C1—C5	−174.7 (6)	C14—N4—C10—C11	−1.2 (9)
C3—N2—C2—C1	2.0 (10)	Pd1—N4—C10—C11	−177.5 (4)
C3—N2—C2—C10	176.8 (6)	C14—N4—C10—C2	−177.7 (5)
N1—C1—C2—N2	−0.6 (9)	Pd1—N4—C10—C2	6.0 (7)
C5—C1—C2—N2	171.4 (6)	N2—C2—C10—N4	129.4 (6)
N1—C1—C2—C10	−174.7 (6)	C1—C2—C10—N4	−56.1 (8)
C5—C1—C2—C10	−2.7 (10)	N2—C2—C10—C11	−47.2 (8)
C2—N2—C3—C4	−1.1 (11)	C1—C2—C10—C11	127.3 (7)
C1—N1—C4—C3	2.7 (10)	N4—C10—C11—C12	1.6 (10)
N2—C3—C4—N1	−1.3 (12)	C2—C10—C11—C12	178.1 (6)
C9—N3—C5—C6	2.8 (8)	C10—C11—C12—C13	−1.0 (10)
Pd1—N3—C5—C6	−170.7 (5)	C11—C12—C13—C14	0.1 (11)
C9—N3—C5—C1	−179.4 (5)	C10—N4—C14—C13	0.3 (9)
Pd1—N3—C5—C1	7.2 (7)	Pd1—N4—C14—C13	176.7 (5)
N1—C1—C5—N3	−135.7 (6)	C12—C13—C14—N4	0.3 (11)
C2—C1—C5—N3	51.9 (8)		

### Hydrogen-bond geometry (Å, °)

**Table:**

*D*—H···*A*	*D*—H	H···*A*	*D*···*A*	*D*—H···*A*
C11—H11···I1^i^	0.95	2.99	3.776 (7)	141.

Symmetry codes: (i) −*x*+1/2, *y*+1/2, −*z*+3/2.
